# The Role of Attention to Emotion in Recovery from Major Depressive Disorder

**DOI:** 10.1155/2013/540726

**Published:** 2013-06-18

**Authors:** Renee J. Thompson, Jutta Mata, Susanne M. Jaeggi, Martin Buschkuehl, John Jonides, Ian H. Gotlib

**Affiliations:** ^1^Department of Psychology, Stanford University, 450 Serra Mall, Bldg 420, Stanford, CA 94305, USA; ^2^Department of Psychology, Washington University in St. Louis, 1 Brookings Drive, Campus Box 1125, St. Louis, MO 63130, USA; ^3^Center for Adaptive Rationality (ARC), Max Planck Institute for Human Development, Lentzeallee 94, 14195 Berlin, Germany; ^4^Department of Psychology, University of Maryland at College Park, 1147 Biology/Psychology Bldg, College Park, MD 20742, USA; ^5^Neuroscience and Cognitive Science Program, University of Maryland at College Park, 2131 Biology/Psychology Bldg, College Park, MD 20742, USA; ^6^Department of Psychology, University of Michigan, 1012 East Hall, 530 Church Street, Ann Arbor, MI 48109, USA

## Abstract

Major Depressive Disorder (MDD) is characterized by several emotional disturbances. One possible but not well-examined disturbance is in attention to emotion, an important facet of emotional awareness. We examined whether attention to emotion predicted recovery from MDD. Fifty-three adults with current MDD completed a week of experience sampling (Time 1). At each prompt, participants reported attention to emotion, negative affect (NA), and positive affect (PA). Approximately one year later (Time 2), the depressive status of 27 participants was reassessed. Participants who had recovered from MDD (*n* = 8) indicated paying less attention to their emotions at Time 1 than did participants who had not fully recovered (*n* = 19). Attention to emotion was better predictor of recovery than was severity of MDD, NA, or PA at Time 1. Levels of attention to emotion at Time 1 in participants who recovered from MDD did not differ significantly from the levels reported by 53 never-depressed individuals who had participated in the experience sampling. Findings indicate that high levels of an otherwise adaptive emotional facet can adversely affect the course of MDD.

## 1. Introduction

Disturbances in emotional experiences are included in the diagnostic criteria for many mental health disorders in the *Diagnostic and Statistical Manual of Mental Disorders* (DSM-5; [1]) and the *International Classification of Diseases* (ICD-10; World Health [2]). For example, heightened levels of negative affect (NA), low levels of positive affect (PA), and excessive guilt are criteria for a *DSM-5* diagnosis of Major Depressive Disorder (MDD). In addition to these diagnostic criteria, individuals with MDD experience greater emotional instability than do healthy controls [3, 4]. Importantly, these wide-ranging disturbances in emotional functioning have been found to impede the adaptive functions of emotions (e.g., effective social interaction; [5]) and to influence the course of mental health disorders [6].

A relatively unexplored emotional disturbance in MDD involves the construct of emotional awareness. We focus on one facet of emotional awareness—attention to emotion, or the extent to which one notices, thinks about, and monitors one's moods [7]. Several theorists have postulated that being aware of one's feelings is vital to being able to use emotional information adaptively [8–10]. Because at least some aspects of the emotional experience of individuals with MDD are, by definition, aberrant from their typical experience (e.g., elevated levels of NA), examining how much they attend to these emotions may provide insight into processes by which MDD is maintained. We examine the extent to which individuals with MDD attend to their emotional experience and whether this predicts the course of the MDD.

Investigators have found that attention to emotion is not related concurrently to levels of depressive symptoms in samples of undergraduate students [7, 11, 12], adolescents [13], and older community residents ([14]; see [7] second sample for an exception). Findings of studies examining the relation between attention to emotion and a diagnosis of MDD suggest a stronger association. Individuals whose MDD was in full remission reported paying marginally more attention to their emotions than did healthy controls [15] but similar levels to individuals who were still depressed [16]. From a different perspective, Saarijärvi et al. [16] also found that decreases in externally oriented thinking (i.e., increases in attention to emotion) were related prospectively to decreases in depressive symptoms in women. (Alexithymia and emotional awareness are made up by two similar underlying dimensions—one of which is attention to emotion; items composing the externally oriented thinking subscale group with items composing attention to emotion scale [10, 17, 18].) In sum, research on attention to emotion in depression has been limited largely to examining either between-group differences in attention to emotion as a function of depression status or within-person comparisons of how levels of attention to emotion predict changes in depressive symptoms. Investigators have not examined whether levels of attention to emotion predict recovery from MDD.

In the present study we used experience sampling method to assess attention to emotion in everyday life during a major depressive episode. We hypothesized that lower levels of attention to emotion would predict recovery from MDD. Central to cognitive therapy for depression is the formulation that negative biases in the perception, interpretation, and recall of information that characterize MDD can lead to emotional experiences that are based on “inaccurate” information [19]. High levels of attention to emotion may be particularly pernicious because emotions influence judgments more strongly in individuals high in attention to emotion than they do in individuals low in attention to emotion [20]. Further, for individuals with MDD, elevated levels of attention to emotion may be maladaptive because there is increasing evidence that lower levels of clarity characterize depression [12, 15], and attending to unclear emotions is likely to be maladaptive. Finally, because MDD has been found to be characterized by low levels of PA and high levels of NA [21], we examined whether PA and NA would predict recovery from MDD more strongly than would attention to emotion.

## 2. Materials and Methods

### 2.1. Participants and Procedure

A total of 106 participants between the ages of 18 and 40 were recruited for a larger project (see [22–24]). All participants were native English speakers. Individuals were recruited from the surrounding communities of Ann Arbor, Michigan, and Stanford, California, via advertisements posted online and at local agencies. The protocol was approved by both universities' Institutional Review Boards. Participants were compensated for their involvement in each portion of the project.

*Baseline Sessions (Time 1)*. At Session 1 participants provided informed consent and completed the Structured Clinical Interview for *DSM*-IV Axis I Disorders (SCID-I; [25]), which assesses both current and past Axis I disorders. Individuals were eligible to participate in the project if they either (1) experienced no current or past mental health disorders (control group; *n* = 53) or (2) were currently diagnosed with MDD (MDD group; *n* = 53). Additional eligibility requirements for the control group included score of 9 or less on the Beck Depression Inventory-II [26, 27]. Participants in the depressed group had to have a BDI-II score of 14 or more and an absence of alcohol/drug dependence in the past six months, absence of diagnoses of Bipolar I or II, and psychotic disorders. Within two weeks of Session 1, participants completed Session 2, which included a series of self-report measures (including the one described later), computer tasks, and instructions regarding the experience sampling protocol. Following Session 2, participants completed the experience sampling protocol (described later) for approximately one week.

The control group (*n* = 53), which was composed of 67.9% women, on average was 25.4 years old (*SD* = 6.4 years) and about half (53.8%) had earned at least a bachelor's degree. The control group was ethnically diverse with 62.3% white, 17.0% Asian American, 9.4% African American, 9.4% multiracial, and 1.9% Latino/a. The MDD group was composed of 71.7% women. The MDD group (*n* = 53), which was composed of 71.7% women, on average was 28.2 (*SD* = 6.4) years old, with about half (50.9%) having earned at least a bachelor's degree. The group was ethnically diverse: 73.6% white, 3.8% Asian American, 5.7% African American, 9.4% multiracial, 3.8% Latino/a, and 3.8% indicating “other.” Information about the participants from the MDD group who participated in the one-year follow-up session is presented next.

*Follow-Up Session (Time 2).* Approximately a year later, the initially depressed participants were invited to complete a follow-up session, which included a diagnostic interview (SCID-I), self-report measures, and computer tasks. Twenty-seven of the 53 original participants completed this session, which occurred 13.8 to 18.9 months after their experience sampling period (*M* = 14.7 months; *SD* = 1.0). Importantly, these 27 individuals did not differ from the 26 initially depressed participants who did not complete the follow-up assessment in severity of initial depressive episode, *t*(51) = .42, *p* = .67, or in demographic variables, including age, *t*(51) = .47, *p* = .64; race/ethnicity, *χ* ^2^(5) = 9.43, *p* = .09; educational level, *t*(51) = .43, *p* = .67; and gender, *χ* ^2^(1) = 2.60, *p* = .11. The mean age of the 27 follow-up participants was 28.6 years (*SD* = 6.7). Twenty-two of the participants were females, and the ethnic/racial composition was 63% white, 15% multiracial, 11% African American, 7% Latino/a, and 4% “other.” Eight of these 27 initially depressed participants were fully recovered from MDD (i.e., eight consecutive weeks limited to only mild symptoms; [28]) at the 12-month follow-up session. The time passing between the initial and follow-up sessions did not significantly differ by recovery status. Antidepressant medication (i.e., selective serotonin reuptake inhibitor, serotonin norepinephrine reuptake inhibitor, and monoamine oxidase inhibitor) use at the baseline session *χ* ^2^(1) = .52, *p* = .47, or the follow-up session, *χ* ^2^(1) = .01, *p* = .65, did not differ by recovery status. In the following analyses, we compare these eight participants with the 19 initially depressed participants who were not fully recovered at the Time 2 assessment.

### 2.2. Self-Report Measures

*Depression Severity.* At Session 1, severity of depressive episode was assessed using the Beck Depression Inventory-II [26, 27], a self-report measure that contains 21 groups of statements describing various depressive symptoms (e.g., loss of pleasure and suicidal thoughts) assessing symptoms over the past two weeks. This measure has been shown to have good reliability and validity [26, 27]. Cronbach's alpha was .92 for the participants who completed the follow-up session.

### 2.3. Experience Sampling

In the week following Session 2, participants carried a hand-held electronic device (Palm Pilot Z22) for seven to eight days that was programmed using the Experience Sampling Program 4.0 [29]. Participants were prompted (via a tone signal) eight times per day between 10 am and 10 pm. Prompts occurred at random times within eight 90-minute windows per day; thus, prompts could occur between a couple of minutes and almost 180 minutes apart. After participants were prompted, they had three minutes to respond to the initial question on the Palm Pilot; otherwise, the device would hibernate until the next prompt and data for that trial were recorded as missing. Up to 56 trials (prompts) of data were recorded for each participant. The 27 participants in this report responded to a mean of 45.3 prompts (*SD* = 7.4), which did not differ significantly from the number of responses of the 26 depressed participants who did not complete the follow-up session, *t*(51) = 1.28, *p* = .21.

*Attention to Emotion.* At each prompt, participants reported the extent to which they were attending to their emotions at the time of the prompt by responding to the item, “I am paying a lot of attention to how I feel right now.” This item was always presented before any affect items and was adapted from the Attention to Feelings subscale of the Trait Meta-Mood Scale (TMMS; [7]). This item was selected for inclusion in the experience sampling protocol because it had the highest factor loading on the Attention to Feelings subscale [7]. Using a 4-point scale (*not at all = 1, little = 2, much = 3, a great deal = 4*), participants indicated at each prompt the extent to which they were attending to their emotions. Individuals' responses over the experience sampling week were averaged to yield a mean score for attention to emotion.

*Affect Ratings.* At each prompt, participants also reported their current levels of negative and positive affect. Using a 4-point scale (*not at all = 1, little = 2, much = 3, a great deal = 4*), participants indicated the extent to which they were currently feeling each of seven negative emotions (sad, anxious, angry, frustrated, ashamed, disgusted, and guilty) and four positive emotions (happy, excited, alert, and active). The emotions were drawn from the Positive Affect Negative Affect Scale [30] and Ekman's basic emotions [31]. Cronbach's alphas were .93 for NA and .80 for PA across aggregated responses to each scale's items. Individuals' responses over the experience sampling week were averaged to yield mean NA and PA scores.

## 3. Results

Before examining our central question, we examined the relations between the predictors, several of which were significantly correlated as would be expected (see Table 1). We conducted a logistic regression analysis to test our central hypothesis that attention to emotion assessed at Time 1 would predict MDD recovery at Time 2, an average of 14 months later. We also tested whether the other predictors included in the study, NA, PA, and depression severity, at Time 1 predicted MDD recovery at Time 2. The size of our data set suggested that the inclusion of one predictor in the logistic regression was most appropriate [32]. Consequently, we ran five logistic regressions including only one predictor at a time. Attention to emotion yielded the largest odds ratio (OR = 8.75, *β* = 2.17, *p* = .04), followed by NA (OR = 7.65, *β* = 2.04, *p* = .09), PA (OR = 2.00, *β* = .69, *p* = .64), and BDI severity (OR = 1.03, *β* = .03, *p* = .55). Although the sample size would prohibit direct comparison of the predictors in a single model, attention to emotion was the only predictor that is significant.

Next, we compared levels of attention to emotion at Time 1 reported by the participants who were depressed at Time 1 and who participated in the Time 2 assessment with levels of attention to emotion reported by the 53 healthy controls who also completed the experience sampling protocol at Time 1 [22]. After removing one outlier who was three standard deviations above the mean for the control group, we conducted a one-way analysis of variance (ANOVA) on level of attention to emotion at Time 1 by diagnostic group (healthy controls, initially depressed individuals who were recovered from MDD at Time 2, and initially depressed individuals who were not recovered from MDD at Time 2). This analysis yielded a significant main effect for diagnostic group, *F*(2,76) = 6.23, *p* < .01. Planned contrasts revealed that the initially depressed individuals who were not recovered at Time 2 (*M* = 2.20, *SD* = 0.55) had significantly higher levels of attention to emotion during the experience sampling week at Time 1 than did both the healthy controls (*M* = 1.88, *SD* = 0.46), *t*(76) = 3.29, *p* < .01, and the initially depressed individuals who had fully recovered from MDD at Time 2 (*M* = 1.76, *SD* = 0.41), *t*(76) = 2.67, *p* < .01. Importantly, those who were fully recovered from MDD at Time 1 and healthy controls did not differ significantly in their levels of attention to emotion at Time 1, *t*(76) = 0.65, *p* = .52.

## 4. Discussion

In this study we examined the role of attention to emotion in predicting recovery from MDD. Individuals who paid more attention to their emotions while in a major depressive episode were less likely to be fully recovered from MDD 14 months later than were individuals who paid less attention to their emotions. This finding is consistent with research examining self-focused attention, a broader construct that has also been implicated in depression (see [33, 34] for reviews). Self-focused attention is part of a self-regulatory cycle involving goal pursuit that leads to high levels of NA when individuals do not attain their standards [35, 36]. Consistent with existing research [37], we found in the present study that attention to emotion was highly related to NA and PA.

We hypothesized that high levels of attention to emotion for depressed individuals would be problematic for two theoretical reasons. First, emotional reactions are affected by individuals' cognitive biases, and second, depression is associated with lower clarity of emotions. It is also possible that higher levels of attention to emotion may be problematic for depressed individuals because of their difficulties with emotion regulation [38]. Lischetzke and Eid [39] found that higher levels of attention to emotion were associated with better well-being in individuals who were effective in regulating their moods but were associated with poorer well-being in individuals with lower mood-regulation scores. It will be important in future research to examine more explicitly and systematically the mechanisms through which attention to emotion is related to recovery from MDD.

It is noteworthy that while the levels of attention to emotion of depressed individuals who subsequently recovered from MDD did not differ from those of healthy controls, higher levels of attention to emotion in depressed individuals predicted a poorer course of MDD. The mean levels of attention to emotion healthy group and those who recovered from MDD suggest that paying some, but not too much, attention to one's emotions is adaptive. We expect it is likely that paying too little attention to one's emotions is maladaptive. In fact, self-focus theories contend that self-focus is adaptive in certain conditions (e.g., self-focus proceeding positive events; [34]). In their mood-as-information theory, Schwarz and Clore [40] posited that emotions are systems through which people receive feedback; when individuals ignore this information, they cannot respond appropriately.

We should note three limitations of this study. First, there was a relatively high rate of attrition from Time 1 to Time 2. Nevertheless, participants who completed the follow-up session did not differ from individuals who did not complete the follow-up assessment in their frequency of responses to the experience sampling prompts, initial depression severity, or any demographic variables. Second, definitions of attention to emotion often include how much individuals monitor and value their emotions. Our single-item measure of attention to emotion did not assess the latter construct. It will be important for future research to assess valuation of emotion and to examine the extent to which individuals are guided by their emotions. Finally, because this study was limited to MDD, additional research is needed to investigate the transdiagnostic role of attention to emotion.

Despite these limitations, there are several strengths of this study, most notably the use of experience sampling to prospectively predict recovery from a carefully diagnosed episode of clinically significant depression. This method minimizes retrospective biases in reporting. Further, whereas measures of attention to emotion that are commonly used require individuals to report on the extent to which they *think* that they pay attention to their emotions, our experience sampling protocol asked participants to report on their level of attention to emotions in real time at a particular moment.

## 5. Conclusions

Kring [41] outlined the importance of examining the interaction of cognition and emotion in increasing our understanding of various forms of psychopathology. We demonstrated in the present study that levels of attention to emotion assessed in a real-world setting predicted a 14-month outcome of MDD, a disorder associated with a variety of emotional disturbances. Thus, this research contributes to our understanding of MDD by indicating that too much of an otherwise adaptive facet of emotional regulation can adversely affect the course of a debilitating disorder.

## Figures and Tables

**Table 1:** Means and correlations of constructs of interest.

	Attention to emotion	NA	PA	Depression severity
Attention to emotion		0.67**	0.45*	0.43*
NA			0.03	0.56**
PA				0.08

Mean	2.14	1.97	1.68	32.7
*SD*	0.57	0.56	0.31	9.4

Note. NA: negative affect; PA: positive affect. *p < .05, **p < .01.
